# Psychiatric service dogs reduce loneliness and improve social functioning in adults with mental health conditions: a qualitative study from South Africa

**DOI:** 10.3389/fpsyt.2026.1958250

**Published:** 2026-10-21

**Authors:** Jacqueline Hoare, Fulufhelo Netshifhefhe

**Affiliations:** 1 Department of Psychiatry and Mental Health Cape Town, University of Cape Town, Cape Town, South Africa; 2 Neuroscience Institute, Faculty of Health Sciences, University of Cape Town, Cape Town, South Africa

**Keywords:** human-animal bond, loneliness, mental health, psychiatric service dogs, qualitative research, reflexive thematic analysis, social functioning, South Africa

## Abstract

**Introduction:**

Loneliness and impaired social functioning are clinically significant features of mental illness. Psychiatric service dogs (PSDs) are individually trained to perform tasks that mitigate the functional impact of psychiatric disability and have been proposed as an intervention for loneliness and impaired social functioning for their handlers living with mental illness. However, the mechanisms through which they may reduce loneliness and improve social functioning are poorly understood and evidence from low-middle income contexts is almost entirely absent.

**Methods:**

This qualitative study aimed to explore the experiences of PSD handlers in South Africa regarding the impact of their PSD on loneliness, social functioning, and psychosocial well-being across a diverse range of mental illnesses. PSD handlers were recruited through purposive sampling via three South African PSD training organisations. Data were collected through semi-structured individual interviews and analysed using reflexive thematic analysis following the six-phase framework described by Braun and Clarke.

**Results:**

Reflexive thematic analysis of participants' accounts generated six themes describing the psychosocial impact of PSD partnership. Three themes capture the primary mechanisms through which PSD partnership improved social functioning and reduced loneliness: (1) alleviation of loneliness through non-judgmental companionship; (2) PSDs as social catalysts: from brief encounters to sustained relationships; and (3) gains in independence and expanded community participation. Two additional themes describe internal psychological mechanisms: (4) restoration of purpose, meaning, and structured routine; and (5) caregiving as a mechanism of self-regulation and re-engagement. A sixth theme addresses the social costs and challenges of PSD partnership: (6) access discrimination, public scrutiny, and the burden of visibility.

**Discussion:**

Together, these themes describe a multi-layered set of psychosocial processes through which PSD partnership both expanded participants' social worlds and imposed new burdens upon them. The findings suggest that PSDs may serve as a meaningful adjunctive intervention for loneliness and social isolation in adults with mental illness. Mental health professionals would benefit from greater awareness of PSDs and the psychosocial benefits and challenges associated with their use.

## Introduction

Social connectedness is widely recognized as important for emotional well-being and mental health (1). The consequences of social disconnection extend well beyond subjective distress; in a meta-analytic review spanning 70 studies, actual and perceived social isolation were both associated with increased risk for early mortality (2). Notably, this elevated risk was found to be comparable in magnitude to that conferred by obesity, underscoring loneliness as a serious public health concern rather than a peripheral psychosocial complaint (2). Loneliness can lead to overactivation of the hypothalamic-pituitary-adrenal axis which is implicated in mental and metabolic disease (3). In addition, loneliness and impaired social functioning are common and clinically significant features of psychiatric illness in adults, cutting across diagnostic categories from posttraumatic stress disorder (PTSD) to depression, anxiety, and psychotic-spectrum disorders (4). These conditions can, in turn, lead to further social isolation and propel a vicious cycle of chronic illness (5). Exploring interventions that can reduce loneliness given its role in the aetiology of the most prevalent chronic diseases of our time is vitally important (5).

Dogs have become tightly woven into the cognitive, emotional, and social fabric of human life (6). Dogs and other companion animals can serve as important sources of social support, providing many positive psychological and physical benefits for their owners (7). Dogs could be a catalyst for several dimensions of human social relationships, ranging from incidental social interaction and getting to know people, through to formation of new friendships (8). A hypothesis in the literature is that dogs can function as “social catalysts,” facilitating interpersonal contact that might not otherwise occur (9). Observational work has found that the presence of a dog is associated with increased positive non-verbal behaviours such as smiling and greater social approachability (9, 10). Walking with a dog may contribute to seeking and enjoying health and fitness, providing purpose and confidence, mobile companionship and a peaceful and contemplative shared respite from everyday life (11). In veterans with PTSD who adopted a dog, close bonds of companionship with their dog increased social interactions, and improved emotional well-being (12). For many pet owners, their pets facilitate relationships from which they derived tangible forms of social support, both of a practical and emotionally supportive nature (8). A qualitative study of older adults who are pet owners found that pets provided comfort and safety, facilitated social inclusion and participation, provided purposeful routine and structure and a meaningful role (13). As older adults can perceive pets as important for their health and wellbeing, findings from a systematic review suggested that community healthcare organisations should develop guidelines and tools for care workers to improve care at home for clients with pets (14). The presence of animals can also significantly impact the health and wellbeing of care home residents with animals providing respite from loneliness, institutionalisation, and illness (15). A systematic review found interacting with animals offered care home residents a way to maintain a sense of self in the care homes, and with support, residents with dementia could also express their identities (15).

Animal assisted interventions with dogs have been effective for reducing loneliness in people who are hospitalized for the treatment of mental illness (16). In a 2015 systematic review exploring the impact of animals on loneliness there was promising evidence that service animals may alleviate social disconnection (17). Against this backdrop, psychiatric service dogs (PSDs) have emerged as a complementary intervention for adults with psychiatric disabilities (18). PSDs are distinguished from companion dogs, emotional support and therapy animals in that they are specially trained to do work or perform tasks directly related to a psychiatric disability, thereby allowing them legal public access rights (19). These tasks may include reducing anxiety through tactile stimulation, nudging, or pawing to help ground the handler in the present moment (18) They may interrupt undesirable behavioural states such as anger outbursts and provide constant body contact or deep pressure stimulation (18). Much of the early PSD evidence base concerns PTSD specifically, however PSDs are increasingly used across a broader range of mental health conditions. PSDs can be task-trained to assist those with panic disorders, anxiety disorders and major depression for example (16, 18).

Beyond facilitating contact with strangers, evidence suggests PSDs may directly reduce subjective loneliness through symptom mitigation that enables greater participation in social and community life. In a non-randomized controlled trial of veterans with PTSD, those partnered with a PSD showed reduced PTSD symptoms and improved psychosocial functioning compared with veterans receiving usual care alone (20). Veterans with service dogs had better emotional health and found social interactions more pleasant; they also had more activity participation but were less likely to leave home (20). This could be due to public stigma, suggesting education on service dog etiquette is needed (21). Qualitative literature also documents social costs including stigma, unwanted attention, and access discrimination that complicate any straightforward narrative of social benefit (22).

Taken together, the literature suggests a plausible but incompletely characterized relationship between PSD partnership and improvements in loneliness and social functioning among adults with mental illness. Multiple mechanisms could be possible including a social catalyst effect that increases positive contact with strangers, direct attachment with the PSD as a buffering of subjective loneliness, and symptom mitigation that enables greater participation in social and community life. At the same time, much of the evidence is drawn from studies in the USA and veteran samples with PTSD, limiting generalizability to the broader population of adults with diverse psychiatric diagnoses. Their applicability to low- and middle-income countries, due to potential lack of accessibility and affordability remains unknown. In addition, service dogs in general are new to South Africa with the first diabetic service dog trained in 2017 and Psychiatric service dogs introduced more recently. The Association of Assistance Dogs SA (AADSA) was founded in 2023 and serves as a national coordinating body for accredited assistance dog training organizations, rather than a direct service provider. AADSA describes its member organizations as upholding rigorous training and welfare standards and promotes internationally recognised best practices in assistance dog training, welfare, handler education and lifelong service dog support.

The current study aimed to conduct semi-structured individual in-depth interviews with handlers who have partnered with a PSD and have a psychiatric diagnosis diagnosed by a mental health professional. The study is complemented by the perspectives of professional PSD trainers on the training, implementation, and broader functioning of PSD partnerships in this context. The present qualitative study seeks to explore the impact of PSDs in South Africa on loneliness and psychosocial functioning for their handlers across a diverse range of mental health disorders. The study aims to inform both mental health care providers and people living with mental illness of the potential impact of partnership with a PSD on loneliness and social functioning.

## Methods

### Participants

Following approval from the University of Cape Town HREC (the approval number 655/2025) the researchers contacted three organizations dedicated to training dogs as guide dogs and service dogs in South Africa: Paws4Life, Honey’s Garden, and Smart Service Dog. These organizations were asked to assist with the recruitment of potential participants by distributing the study’s advertisement to individuals affiliated with their training programmes who are currently handlers of PSDs.

The recruitment advertisement outlined the purpose of the study and detailed the inclusion criteria for participation. To be eligible, participants were required to have a mental health condition diagnosed by a qualified mental health professional and currently be the handler of a PSD that has been actively working for at least six months. The latter criterion was included to ensure that participants had sufficient time to establish a meaningful relationship with their PSD and to experience its potential social benefits. Participants are aged 18 years or older, capable of providing informed consent, participating in an interview and able to understand and communicate in English. Additionally, participants could be receiving psychiatric treatment (e.g., medication or psychotherapy) or not currently engaged in formal mental health services. A secondary, purposively sampled group of the three organizational leads in the three partnering training organizations and directly involved in training PSDs for clients with mental health conditions, was also recruited. They provided contextual and professional insight into PSD support, PSD training, functioning, and the factors influencing successful or unsuccessful partnerships, complementing rather than substituting for handlers’ own accounts.

Individuals who were interested in participating contacted the research team via email to express their interest in the study. Eligible participants were subsequently provided with an information sheet about the study and a consent form. Once written informed consent had been obtained, an interview was scheduled. Participants were informed that their participation was voluntary and that they could withdraw from the study at any time without consequence. Purposive sampling was employed to identify participants who met the study’s inclusion criteria and who had lived experience of being a handler of a PSD. The researchers aimed to interview 15–20 participants to reach data saturation.

### Interviews

PSD handler interviews were all conducted by KN a psychiatric registrar in training at UCT. Organizational lead interviews were conducted by JH a senior consultant psychiatrist at UCT. Each interview lasted approximately 45 to 60 minutes. Interviews were conducted either online or in person, depending on the participant’s preference and proximity to Cape Town. Online interviews were conducted via a University of Cape Town Teams account and in person interviews were conducted in the Neuroscience Institute at the University of Cape Town. All interviews were audio recorded and transcribed verbatim for analysis. The interviews were guided by a semi-structured interview schedule designed to explore participants’ psychosocial and well-being benefits associated with PSD partnership. PSD handler participants were asked a series of open-ended questions, including:

- Can you describe what led you to become a PSD handler?
- What were your personal goals or expectations when you decided to partner with a PSD?
- How, if at all, has your mental health changed since partnering with your PSD?
- Aside from your PSD partnership, what other mental health treatments have you used or are you currently using (e.g., therapy, medication)?
- How have relatives, friends, and healthcare professionals responded to your service dog?
- How do you experience access to shops, public services, or other public spaces when accompanied by your service dog?
- Has your service dog affected your social interactions?
- What has been the most helpful aspect of having a PSD?
- What tasks has your service dog been trained to perform?
- What challenges or drawbacks have you experienced as a handler of a PSD?
- Has the presence of your service dog alleviated any symptoms? If so, which symptoms and in what ways?
- What do you see as the limitations of having a PSD?

These questions were asked in the same order and served as prompts to initiate discussion and to provide a flexible structure for the interviews. Additional follow-up and clarification questions were asked during the interviews to gain deeper insight into participants’ experiences and to ensure an accurate and comprehensive understanding of their perspectives as PSD handlers. The use of clarification and probing questions also facilitated rapport building and encouraged participants to elaborate on their experiences in greater depth. Following the completion of an interview, participants received a token of appreciation from *Chommies*, a Cape Town–based company that manufactures and sells dog accessories. Each PSD handler participant received a dog leash valued at R750. The leash was not intended to influence participation or responses.

Organizational leads were asked to reflect on their experiences of training psychiatric service dogs and partnering them with potential handlers living with mental illness in South Africa. They were asked to share their insights on what makes a successful PSD and PSD handler partnership and which factors may lead them to say that partnering with a PSD may not be recommended.

### Data analysis

Data were analysed using reflexive thematic analysis, employing inductive and semantic methods, as described by Braun and Clarke. Reflexive thematic analysis was selected because it centres the active, interpretive role of the researcher in the construction of meaning from qualitative data. It is well suited to exploratory research with the aim is to illuminate the range of lived experience rather than to test predetermined categories (23, 24). Within this approach themes are understood as researcher constructions and developed through sustained, reflexive engagement with the data (23, 24). The analysis followed the six phases outlined by Braun and Clarke (23).

The second author (F.N.), who also conducted all PSD handler interviews, served as the primary analyst throughout the analytic process. This approach was considered appropriate within a reflexive thematic framework, as her sustained immersion in the data from the point of data collection supported deep familiarisation and enabled her to bring nuanced contextual knowledge to the interpretive process. The first author (J.H.), who conducted the organisational lead interviews, engaged with the analysis as a collaborative reflexive partner throughout, contributing additional interpretive perspectives, challenging emerging interpretations, and co-developing the final thematic structure. This collaboration is understood within the reflexive thematic framework not as a reliability-checking procedure but as a generative reflexive dialogue in which two researchers brought different positionalities and analytic sensibilities to bear on the data.

The second author engaged in repeated reading of the interview transcripts to achieve thorough familiarisation with the data (23–25). Computer-assisted notetaking and highlighting were utilised to capture initial impressions. The aim of this phase was not to identify codes, but to develop a rich, holistic sense of each participants’ account before any systematic coding was undertaken (25). The second author, then generated initial codes by identify meaningful segments of text across the dataset that were relevant to the research questions (23–25). Coding was conducted manually, and codes were applied across the entire dataset. Codes were understood as research generated labels, capturing what was meaningful in the data relative to the research aims. At the conclusion of this phase, the second author shared her coded transcripts and an overview of her initial coding with the first author. The first author engaged with this material reflectively, discussing interpretive decisions with the second author and offering alternative readings, were relevant as a means of deepening and complicating the emerging analysis rather than verifying its accuracy. The codes identified were then examined for patterns and grouped into broader categories (23, 25). Related codes were collated to form potential themes that captured the participants lived experiences as handlers of PSDs. The themes identified were subsequently reviewed and refined by both researchers (24, 25). Both researchers examined all coded data extracts to ensure that they formed coherent and meaningful patterns within each theme (24, 25). Following the review process, each theme was further refined, clearly defined, and appropriately named to capture its central meaning by both researchers. This phase involved identifying the essence of each theme and determining how it related to the overall research question. This process ensured that each theme accurately reflected the underlying data and contributed meaningfully to the overall narrative of participants’ experiences (25). The final themes were organised into a coherent narrative, supported by illustrative quotes from participants.

Throughout the analytic process, reflexivity was maintained to acknowledge the researcher’s perspectives and potential influence on the interpretation of the data (24, 25). The researchers engaged in ongoing reflection to ensure that the findings remained a reflection of the participants’ accounts rather than being shaped by preconceived assumptions (25). Reflexivity was enacted through several concrete strategies. The second author maintained a reflexive journal throughout data collection and analysis, in which she recorded observations about her own reactions to interview content, emerging interpretive inclinations, and moments of uncertainty or discomfort during coding. For example, early in the analytic process she noted a tendency to code accounts of participants’ emotional connection with their PSD as straightforwardly positive and recorded a prompt to interrogate whether this reflected participants’ own framings or her own affirmative assumptions about the human-animal bond. Returning to the raw transcript extracts rather than her initial coded summaries were used as a corrective strategy when such tendencies were identified. Regular reflexive discussions were held between both authors at key analytic junctures, including after initial coding, after the generation of candidate themes, and during theme review.

Familiarity with the PSD literature and PSD context in South Africa was actively treated as a source of potential bias rather than simply as interpretive context. At the theme development stage, both researchers explicitly discussed whether the generated themes were genuinely grounded in the data or whether they had been inadvertently shaped by theoretical constructs already present in the literature, such as the social catalyst hypothesis.

## Results

Twenty-two participants consented to participate in the study. Of these, 19 participants were PSD handlers and three were leads of organisations involved in the training of PSDs. Data from all 22 interviews were included in the qualitative analysis. The inclusion of the organisational leads provided additional contextual and experiential insights into the training, implementation, and broader understanding of PSDs in SA. The participants included 15 females, 3 males and one non-binary, with ages ranging from 21 to 51 years old. The majority of participants had been diagnosed with more than one mental health condition, including depressive disorders (n = 11), anxiety disorders (n = 13), trauma and stressor-related disorders (n = 4), neurodevelopmental disorders including autism spectrum disorder and ADHD (n = 10), and bipolar and related disorders (n = 6). Twelve participants reported comorbid medical conditions, 16 were receiving pharmacological treatment, and 14 were engaged in psychotherapy or occupational therapy at the time of interview (See **Table 1**). Treatment for mental health conditions included pharmacological interventions such as antidepressants, antipsychotics, mood stabilisers, stimulants, and benzodiazepines. The PSDs ranged in age from 1 to 9 years, and the duration of time the dogs had been working as PSDs ranged from 10 months to 5 years.

**Table 1:** Demographics and characteristics of the PSD handler participants.

Variable	n	%
Gender		
Men	3	15.8
Women	15	78.9
Non-binary	1	5.3
Age		
18–21 yrs	2	10.5
22–36 yrs	10	52.6
37–54 yrs	7	38.8
Race		
White	18	94.7
Black	1	5.3
Marital status		
Married	5	26.3
Single	12	63.2
Divorced	0	0
Separated	1	5.3
Widowed	1	5.3
Education level		
High school	7	36.8
Bachelor’s degree	3	15.8
Honor’s degree	4	21.1
Master’s degree	4	21.1
PhD	1	5.3
Employment status		
Employed	8	42.1
Self-employed	5	26.3
Unemployed	6	31.6
Place of residence		
Within Cape Town	11	57.9
Outside Cape Town	8	42.1
Living condition		
Lives alone	3	15.8
Lives with family/friends	16	84.2
*Mental health condition		
Depressive disorders	11	57.9
Anxiety disorders	13	68.4
Trauma and stressor-related disorders	4	21.1
Personality disorders	2	10.5
Neurodevelopmental disorders (ASD, ADHD, Learning disorders)	10	52.6
Bipolar and related disorders	6	31.6
Eating disorders	1	5.3
Obsessive-compulsive disorders	1	5.3
Comorbid medical conditions		
Yes	12	63.2
No	7	36.8
Receiving medication		
Yes	16	84.2
No	3	15.8
Attending therapy (occupational, psychotherapy, physiotherapy and others)		
Yes	14	73.7
No	5	26.3

*The majority of participants in the study had been diagnosed with multiple mental health conditions.

### Overview of themes

Reflexive thematic analysis of PSD handler participants’ accounts generated six themes describing the psychosocial impact of PSD partnership. Three themes capture the primary mechanisms through which PSD partnership improved social functioning and reduced loneliness (1): alleviation of loneliness through non-judgmental companionship (2); PSDs as social catalysts: from brief encounters to sustained relationships; and (3) gains in independence and expanded community participation. Two additional themes describe internal psychological mechanisms (4): restoration of purpose, meaning, and structured routine; and (5) caregiving as a mechanism of self-regulation and re-engagement. A sixth theme addresses the social costs and challenges of PSD partnership (6): access discrimination, public scrutiny, and the burden of visibility. Together, these themes describe a multi-layered set of psychosocial processes through which PSD partnership both expanded participants’ social worlds and imposed new burdens upon them.

#### Theme 1: alleviation of loneliness through non-judgmental companionship

The most consistently described psychosocial benefit of PSD partnership was a direct reduction in subjective loneliness, experienced through the constant, unconditional presence of the PSD. This was particularly salient for participants who lived alone or whose mental health conditions had substantially eroded their human support networks. Unlike human relationships, the companionship provided by the PSD was described as free of judgment, expectation, and the emotional labour that participants often found exhausting in interpersonal interactions. Participants did not simply describe feeling less alone; they described a qualitatively distinct form of relational presence that they experienced as uniquely available and unconditionally accepting.

One participant, who described her emotional support network as inadequate for her needs and her living situation as isolating, captured this directly: *“She has definitely alleviated feelings of disconnection and loneliness … she’s a constant companion.”* Another described the significance of having a living being attend to her emotional state: *“Just the fact of not being alone … and noticing how he cares”*.

This non-judgmental quality of the relationship was experienced as particularly meaningful by participants who frequently felt scrutinised or misunderstood by others in the context of their mental health conditions. One participant, who described the relational texture of her bond with her PSD in detail, explained: *“Most helpful is just having her presence with me. She is my companion. She really keeps me calm. And breathing together with her if I’m feeling overwhelmed at all.”* The nature of this companionship physical closeness and shared breathing was described as a mechanism of emotional co-regulation that operated independently of verbal communication or the reciprocal demands characteristic of human relationships.

The attunement of the PSD with the handler’s emotional state was also experienced as a form of being known and witnessed, which itself reduced isolation. One participant described this responsiveness during a heated work meeting: *“He can pick up very well on what’s going on with me. So even if a meeting gets heated, he will come to me, comfort me and be more affectionate.”* Another described how her PSD had been trained to respond to physiological cues of anxiety *“to pick up on signs of anxiety, so just any kind of leg jiggling, heavy breathing” illustrating* how her PSD’s trained responsiveness created an experience of being consistently and accurately perceived, which appeared to meaningfully offset the subjective sense of disconnection.

#### Theme 2: PSDs as social catalysts — from brief encounters to sustained relationships

While Theme 1 describes the PSD’s direct buffering of loneliness through companionship, a second and distinct psychosocial process emerged in participants’ accounts: the PSD’s capacity to catalyse social interactions with other people that would not otherwise have occurred. This social catalyst function operated across a gradient, from brief, incidental encounters in public settings, through the gradual rebuilding of social confidence, to the development of sustained friendships and community connections.

##### Initiating social contact in public settings

Participants consistently described how the presence of their PSD in public created organic, low-stakes opportunities for brief social contact that functioned as entry points into broader engagement. The PSD lowered the threshold for interaction with strangers, making social contact feel less threatening and more manageable. One participant described this process with precision: *“Because he needs exercise, I need to go walking with him. And inevitably, when we walk, somebody will comment about how he looks. I’ve learned that it’s okay if I need to go and have a breather and take a walk somewhere, and I’m more than capable of talking to others. In the past, I would have been too scared”*.

Another participant noted: *“Somebody will comment, so you have a quick chat, which I normally would not have done.”* These interactions, although brief, were described as significant precisely because they occurred at all, moments of human contact that the participant’s anxiety would previously have foreclosed. The PSD effectively externalised the handler’s social identity, giving others a non-threatening and positive point of contact that drew attention away from the handler’s own distress or self-consciousness.

##### Rebuilding social confidence through graduated exposure

An important and distinct process described by participants was the role of these repeated, PSD mediated interactions in gradually rebuilding social confidence. For participants with social anxiety, the PSD functioned as a social scaffold, present enough to make interactions feel manageable, but not so intrusive that the handler remained entirely dependent upon the PSD as an intermediary. One participant with social anxiety and ADHD described this trajectory explicitly: *“ The mere fact that he’s there and that I can sort of pet him, smell him, that kind of thing, that sort of gives me a little bit more confidence. Having the confidence to speak to somebody that I don’t know and being the platform on which I can to speak to strangers”*.

This account illustrates a key developmental dynamic: the PSD’s presence enabled initial contact, but over time, the handler internalised the capacity for social engagement, reducing dependence on the dog as a crutch. Another participant described a similar shift: *“It helped my social anxiety immensely … it’s showing me that not everybody’s a threat.”* The repeated experience of positive, non-threatening social contact appeared to recalibrate participants’ social expectations over time.

##### From superficial contact to meaningful relationships

Participants described how the brief social encounters catalysed by their PSD partnership could over time develop into sustained friendships and broader community connections, a process that extended well beyond the PSD’s immediate facilitative role. One participant described this transformative effect in detail: *“I’ve made so many friends because of him … he’s really opened doors for me that before would never have opened”*.

#### Theme 3: restoration of purpose, meaning, and structured routine

A third distinct psychosocial mechanism described by participants concerned the restoration of a sense of purpose and meaning in daily life through the responsibilities of PSD partnership. For many participants, psychiatric illness had eroded the structures, commitments, and roles that ordinarily confer meaning, including employment, relationships, and participation in community life. The PSD introduced a new and compelling source of purpose that was not contingent on mental health status or social approval, and that operated even on the most difficult days.

Participants described the responsibility of caring for the PSD as providing an external source of meaning that persisted even when their internal motivation had collapsed. One participant articulated this in terms that map directly onto the clinical literature on meaning and purpose as therapeutic goals for people with mental health conditions: *“The training gives you purpose in what can sometimes feel like a purposeless situation. It’s really important to be responsible for something other than just yourself … it gives you purpose”*.

This sense of purpose was closely linked to the establishment of daily routine. Participants described how the consistent biological needs of their PSD to be fed, walked, and attended to, imposed a structure on the day that was experienced as regulating, particularly during depressive episodes when self-care and daily functioning had deteriorated. One participant described this dynamic with notable clarity: *“If I’m in a depression phase, I need to get up to take him to eat. It makes a big difference, because normally I would just stay in bed and not get up for days. Where now, I can’t do that because where is he gonna pee? So it’s a bit of a guilt trip thing from the dog, because one of his tasks is to bring me my medication. He can’t force you to take your meds, but you feel guilty because he brought it to you. But for me, it helped the small stuff, needing to take him out, just standing up when I don’t want to has helped me quite a bit”*.

This account illustrates how a trained task medication retrieval interacted with the affective experience of caregiving responsibility to produce a chain of behavioural activation that would not have occurred without the dog’s prompting. The routine established by the dog’s needs extended to physical activity, with several participants describing how the necessity of walking their PSD had led to sustained engagement in outdoor exercise with one participant describing how the requirement to exercise her PSD had ultimately led her to complete a marathon, a particularly striking example of how purpose cascades into broader life participation.

For participants who trained their own PSDs, the training process itself became a source of meaning and pride. The successful development of the dog’s skills was experienced as an achievement that built confidence and self-efficacy in domains where illness had previously undermined both: *“Being able to train a dog from start to finish gives you a lot of empowerment.”* These findings are consistent with evidence that companion dog ownership gives people with mental health conditions a sense of purpose and meaning and extend this finding by illustrating the specific mechanisms of structured routine, embodied responsibility, and skill development through which purpose was generated and sustained in this sample.

#### Theme 4: gains in independence and expanded community participation

A fourth psychosocial process concerned participants’ descriptions of increased independence not merely as a practical outcome, but as a psychologically meaningful reclamation of agency and autonomy. Prior to PSD partnership, many participants described their access to public spaces, social events, and daily activities as substantially curtailed by anxiety, avoidance, and dependence on others to accompany them. The PSD changed this in ways that participants experienced as profoundly significant. One participant described the experience of entering a shopping centre alone for the first time in four to five years, the day after her PSD qualified: *“We actually went to a mall, and it was the first time I had been inside a mall by myself in, like, four or five years. We were both very stressed. We were both like, ‘Oh, my God, this is crazy.’ We went in and out very quickly, but yeah”*.

The same participant described attending a function shortly afterwards *“if I didn’t have her with me, I would have turned around and left because there were so, so many people” illustrating* how PSD partnership enabled participation in social and cultural life that anxiety had previously made inaccessible. The reduction of dependence on others was experienced as particularly significant. Several participants described having previously required a family member or friend to accompany them to manage their anxiety in public settings; PSD partnership enabled them to navigate these situations alone: *“I just sort of really wanted to improve my mental health and just like being able to go out in public by myself and not freak out or need to rely on other people. So, it was more of like gaining that independence back, and feeling like I had control over myself, if that makes sense.”* Another described the broader significance of this independence as extending beyond practical logistics to a sense of relational autonomy within her family: *“I think it’s the journey I’ve been on for a while on trying to be independent from my family dynamic, and with her, I think she was like the start of the ripple in the water”*.

Participants also described a specific mechanism through which the PSD enabled social navigation in public: by drawing others’ attention and providing a socially acceptable focal point, the dog deflected scrutiny away from the handler: *“I think it has changed in that I’m more comfortable approaching somebody else and I’m more comfortable with somebody else approaching me. They focus on him rather than on me and my insecurities, they don’t really matter because I don’t feel like they’re judging.”* The PSD also enabled handlers to exit social situations that had become overwhelming, with one participant describing a learned signal: *“He’s got a signal that I give to him to get out of a situation”* — and another noting that her dog could locate the nearest exit on command. This capacity to enter and exit public spaces with greater autonomy represented a meaningful expansion of the social world available to participants.

#### Theme 5: caregiving as a mechanism of self-regulation and re-engagement

A fifth psychosocial process concerned the role of the caregiving relationship itself caring for the PSD in interrupting maladaptive psychological patterns and promoting re-engagement with daily life. Participants described how attending to the PSD’s needs redirected attention away from rumination, illness preoccupation, and internal distress, and toward purposeful outward-directed activity. Crucially, this was not experienced as avoidance but as a constructive reorientation. One participant described the interruptive function of the PSD’s presence during periods of psychological overwhelm: *“Sometimes it is really important because you get really tangled up in your own stuff. And joy is such a massive distraction from pain and debilitation.”* Another described the PSD as a grounding focus during sensory overload in public: *“Everything isn’t so overwhelming because I can focus on her. I can just focus on her and get out”*.

A particularly striking pattern across multiple participants was the reciprocal relationship between caring for the PSD and caring for oneself. Meeting the PSD’s basic biological needs feeding, walking and veterinary care created a chain of self-care that participants might not otherwise have initiated. One participant described this explicitly: *“Because I was taking care of something else, and even though I wasn’t taking good care of myself, now I had to take care of myself because I have another person, so to speak, that I have to look after”*.

Another described this chain concretely: *“You know you have to get up in the morning, you have to feed your dog, so if you feed your dog, you need to eat as well.”* This pattern in which the PSD’s needs bootstrapped the handler’s own self-care operated as a mechanism of behavioural activation that was embedded in the affective relationship rather than externally imposed. One participant summarised the reciprocal nature of this dynamic: *“You can’t take care of your dog if you can’t take care of yourself. Those things go hand in hand”*.

#### Theme 6: access discrimination, public scrutiny, and the burden of visibility

While the preceding themes document substantial psychosocial benefits of PSD partnership, participants also consistently described a range of negative experiences that created significant burden and, in some cases, directly undermined the very benefits the PSD was intended to provide. These negative experiences were not peripheral to participants’ accounts; they were described with considerable detail, emotional weight, and frequency, and constituted a distinct and important dimension of the lived experience of PSD partnership in South Africa. Several interconnected processes were evident: access discrimination; intrusive public questioning; the involuntary disclosure of invisible disability and the paradoxical increase in anxiety that public scrutiny could produce.

One participant described the cumulative toll of this experience: *“Fighting with people about access has been the biggest one, because very often people will just be like, ‘No, she can’t come in.’ And when I give them their papers, and then they still try to bully me. And I’m like, ‘Listen, this is not acceptable.’”* Another participant described the emotional and practical management required simply to enter a shop: *“Yes, you want the world to be like, ‘Oh, I know what a service dog is.’ But also you kind of just have to make your life less complicated because you don’t want to fight every day with a store because they didn’t let you in — because it’s tiring. It’s draining. Some days I’m just like, ‘Okay, whatever. I’m going.’ While other days, I will fight you.”* The same participant described keeping a saved copy of the relevant disability legislation on her phone for rapid access during such encounters.

The presence of the PSD frequently prompted intrusive questions from members of the public about the nature of the handler’s disability creating a form of involuntary disclosure that many participants found distressing and exhausting. This was particularly burdensome because the disability was psychiatric and therefore invisible, requiring explanation rather than being self-evident. One participant described the paradox this created: *“It’s ironic that sometimes the reason I have my dog is for anxiety, first and foremost to keep me calm, and then being provoked with intrusive questions and having to hand over my diagnosis, having to explain myself — that can get exhausting and that’s very draining”*.

Several participants described a particular irony: that being in public with their PSD could itself generate the very anxiety it was intended to mitigate, by drawing heightened attention and scrutiny. One participant articulated this tension directly: *“More panic arises when I do go out in a public space because I know that all eyes are on you and you’re representing something that a lot of people have never seen or heard of before”*.

Finally, participants described the additional layer of stigma specific to psychiatric disability. Several noted that some handlers chose to conceal the psychiatric nature of their PSD’s role, or were reluctant to obtain a PSD at all, due to the disclosure of mental illness that it implied. One participant noted: *“A lot of people, particularly with a psychiatric service dog, can be quite ashamed of their mental health conditions, or maybe don’t want to always disclose that to everyone.”* Another, reflecting on the broader sociocultural context in South Africa, observed that admitting the need for psychiatric help remained particularly stigmatised in more conservative cultural communities.

Together, Themes 1 through 5 describe a set of interconnected psychosocial benefits: direct buffering of loneliness, facilitation of social contact and relationship formation, restoration of purpose and routine, expanded independence, and caregiving-mediated self-regulation that operated together to improve social functioning and reduce isolation among participants. Theme 6 documents the significant social costs that accompanied these benefits, and which in some cases actively undermined the benefits of PSD partnership.

### PSD organisational lead accounts

The three organisational leads whose accounts are reported here represent the founders of the three South African PSD training organisations represented in the study sample. Their accounts offer complementary perspectives on training philosophy, handler selection, dog placement, and post-placement monitoring.

#### Psychiatric service dogs training

The organizational leads all described task-based training that is individually tailored to each handler’s specific psychiatric disability rather than following a standardised curriculum. They described the intake process as one of identifying precisely how a dog could fit into each handler’s life and building a task repertoire around that: *“We take their disability or their condition and then we ask, during an interview, we would work out how exactly a dog could fit into that and make their lives easier. And then we create tasks around their conditions, so everyone is completely tailor trained for the person they’re gonna be with”*.

One organizational lead described a fundamental shift in her organisation’s training model following reflecting on failed placements. Her organisation no longer pre-trains psychiatric service dogs and places them with handlers after two to three weeks of transition training. Instead, she now operates exclusively through an owner-training model, in which the handler trains their own dog across a period of two to three years: *“We no longer do pre-trained psychiatric dogs. We only do it via our owner-trained program, which means that that person either has a dog already that they have a relationship with, that they really know. Doesn’t matter if that dog’s already eight or nine years old. As long as they’ve got the ability to pass the tests, and do the tasks, and be in public spaces”*.

One organisation described integrating psychologists directly into the training process, not in a clinical role with the handler, but as part of the training team itself: “*We have four psychologists that are part of our team.”* They also described training dogs specifically to resist environmental distractions as a core training focus, enabling the dog to maintain its working behaviour despite the attention it receives in public spaces.

#### Potential handlers screening prior to PSD partnership

The organizations all described a multi-stage assessment process that went beyond accepting a referral letter from a mental health professional. One lead described conducting an intake interview with prospective handlers, following which she contacts the applicant’s psychologist or psychiatrist directly to provide psychoeducation about the realities of PSD ownership, including the challenges of public attention, the burden of responsibility, and the risk that the dog may not produce the expected benefits for every person. This pre-assessment psychoeducation is framed as essential: *“My focus at the moment is talking to, when I get an application come in, I speak to their psychologist or their psychiatrist, and I explain all of that to the psychiatrist so that they can explain to the person the reality of having a dog with you”*.

She also described her organization’s shift to an owner-training model as itself functioning as a screening mechanism: the requirement that a handler train their own dog over two to three years generates a body of evidence about their real-world capacity to manage the dog before any formal qualification is granted: *“In some ways, by changing the training, you’re doing an assessment of their ability to live with a dog before you take them on as clients, because they have actual evidence or history of being able to do this right”*.

Another lead described a multi-disciplinary team assessment process, with applications reviewed within 48 hours and the organization’s psychologist: *“We have a psychologist meeting once a week, and we’re trying to do it within 48 hours of the application. Because we don’t tell the person, ‘Listen, we’ll get back to you in another month and we have a meeting.’ The person’s already stressed”*.

One organization lead described requiring all handlers to complete a dog first aid course before receiving a dog, as a condition of placement. All organization leads described psychoeducation about the challenges of public access as a standard pre-placement component.

#### PSDs selected for placement with handlers

The organizations described differing approaches to dog selection, reflecting their contrasting training models. One organisation selected dogs on behalf of handlers and had strict criteria around dog suitability, particularly preferring dogs bred or sourced specifically for service work rather than rescue dogs: *“The dogs that we train for guide dogs have to meet a certain criteria. They may not be a rescue dog. We’re trying to limit the fail rate of that dog. We don’t want expectations on the person with a dog that’s already got a lot of luggage”*.

The owner-training model means that handler and dog are already paired prior to formal training, with the pre-existing relationship between handler and dog considered an asset. In cases where a handler does not have an existing dog, the organisation assists them in identifying a suitable dog to train with. All organisations noted that the dog’s temperament, trainability, and capacity to tolerate public environments are assessed during the training process as prerequisites for qualification.

#### Monitoring of the PSD-handler placements

One organisational lead described a formalised monitoring system of the which he characterised as a lifelong support commitment rather than a time-limited intervention: *“We also have to check up on the dog. And it’s about the relationship for the next 15 years. So it’s like a monthly check-in. If you get a dog this month, we’ll check in with you every other day. After two weeks, we go to maybe three days a week. After a month, we’re down to once a week. And then by month four or five, we’re down to once a month. But we’re still monitoring what’s going on”*.

Another described monitoring in terms of managing her teams’ public access experiences, with an explicit commitment to intervening whenever one of her teams encounters an access denial: *“Any time any of our teams get stopped, no matter where they are, they get the details, tell us, and then we tackle the place and we educate as we go. And we hardly have problems anymore”*.

#### Reasons why a PSD-handler placement may not work

Drawing across the three interviews, the following reasons were identified:

Handler-related factors:

- Severity and nature of the psychiatric condition: The leads described certain conditions as incompatible with the demands of PSD ownership, not because of a value judgement about the person, but because of the functional requirements of caring for and working with a trained dog. One stated plainly: *“If you can’t get out of bed most days, you probably can’t have a service dog”*.
- Memory impairment: Handlers who experience cognitive difficulties as part of their condition may forget gear, paperwork, or training protocols, creating both practical and welfare problems.
- Misaligned expectations — the ‘magic wand’ problem: The leads identified a pattern in which prospective handlers imagined the PSD would resolve their difficulties without sufficient appreciation of the responsibilities and challenges involved. One observed: *“People almost imagine this to be a magic wand. Like, if I’ve got my dog with me, I can do anything”*.
- Inability to maintain consistent training and reinforcement: Dogs trained to perform specific behaviours require ongoing reinforcement. If the handler does not respond to the dog’s cues the trained behaviours extinguish.
- Inability to provide good care: The welfare of the PSD was identified by all leads as a non-negotiable condition of placement.

Dog-related factors:

- Dogs carrying prior behavioural problems: One lead described rescue dogs or previously owned dogs with unresolved behavioural histories as presenting a higher risk of placement failure.
- PSDs becoming reactive in response to handler anxiety: One lead shared an account of the dog that became extremely reactive because it was consistently paired with an anxious handler illustrates the bidirectional nature of the dog-handler relationship and the risk that the handler’s symptoms can negatively affect the dog’s behaviour.

Contextual and systemic factors:

- Public access challenges escalating handler stress: The leads acknowledged that the demands of navigating public access discrimination, intrusive questioning, and involuntary disclosure of psychiatric disability could, in some cases, make the PSD partnership more rather than less stressful.

## Discussion

This qualitative study provides experiential evidence that psychiatric service dogs meaningfully address loneliness and improve social functioning among adults with a diverse range of psychiatric disorders in South Africa, a context that is notably absent from the existing evidence base. Three themes captured the primary mechanisms through which PSD partnership improved social functioning and reduced loneliness (1): alleviation of loneliness through non-judgmental companionship (2); PSDs as social catalysts: from brief encounters to sustained relationships; and (3) gains in independence and expanded community participation. Two additional themes describe internal psychological mechanisms (4): restoration of purpose, meaning, and structured routine; and (5) caregiving as a mechanism of self-regulation and re-engagement. A sixth theme addresses the social costs and challenges of PSD partnership (6): access discrimination, public scrutiny, and the burden of visibility. Together, these themes describe a multi-layered set of psychosocial processes through which PSD partnership both expanded participants’ social worlds and imposed new burdens upon them. These findings extend the existing literature and offer theoretical insights into how PSDs may improve social functioning and reduce loneliness in this population.

The finding that PSDs directly reduced subjective loneliness through non-judgemental companionship aligns with and extends the emerging clinical evidence. Participants consistently described their PSD as alleviating feelings of disconnection and isolation, particularly for those living alone, through a form of non-judgmental, relational presence that was experienced as qualitatively distinct from human support. A recent narrative synthesis of 24 qualitative studies of companion animal relationships among people with mental health difficulties identifies non-evaluative companionship as a distinct therapeutic mechanism that companion animals offered support that was non-coercive and non-evaluative, which may be significant as people living with mental health conditions frequently feel scrutinised or assessed rather than supported (26). Companion animals supported social connection through non-demanding companionship that mitigated loneliness without requiring disclosure, emotional labour, or conversational reciprocity (26). Dogs have been conceptualized as affective scaffolds who enable and modulating human experience and the accounts of participants in this study speak to the profound role PSDs can play in the emotional architecture of daily life (6). A qualitative study found that the human–companion dog bond functions as a stable emotional anchor, promoting non-judgmental connection and emotional security, with dogs enhancing psychological safety and emotional expression especially for individuals with limited human support (27). McConnell and colleagues demonstrated experimentally that pets can satisfy fundamental social needs for belonging, self-esteem, meaningful existence, and a sense of control, in ways that are functionally comparable to human social relationships, and without diminishing the quality or importance of human connections (7). Reniers et al. proposed that pets occupy a distinct and valuable position within the social support ecosystem, one that is particularly salient for individuals whose human support networks have been depleted as is common in the context of psychiatric disability, social withdrawal, and marginalisation (14). Our findings together with the findings in studies exploring the human-companion dog bond suggest that PSDs may be source of companionship that directly buffers subjective loneliness.

The social catalyst function of PSDs was one of the most consistently described experiences across participants. Handlers recounted that the presence of their dog created organic, low-pressure opportunities for social contact and brief conversations with strangers that functioned as entry points into broader social re-engagement. This is consistent with evidence that the presence of a dog is associated with increased social approachability and more frequent interactions with strangers in public settings (9, 10). Emerging quantitative evidence also suggests that veterans with service dogs reported higher quality, though not necessarily greater quantity, of social interactions in daily life (21). Orr and colleagues found that animal presence was associated with improved social interaction, reduced loneliness, and enhanced engagement with others and with qualitative studies highlighting that animals created a shared focus of attention that facilitated conversation and social contact among residents in care homes who might otherwise have remained isolated (15). The present findings also suggest that for many participants, these superficial encounters developed over time into sustained friendships and meaningful community connections. Wood et al. demonstrated, in a large mixed-methods community study, that companion animals functioned as a conduit for social interaction, the formation of new friendships, and the development of broader social support networks, with the majority of pet owners reporting that their pet had helped them get to know people in their neighbourhood who they would not otherwise have met (8). This suggests that the social catalyst effect of PSDs may produce both immediate conversational access and longer-term network expansion.

The themes of purpose, routine, motivation, and the caregiving-focused redirection of attention represent pathways to improved social functioning emerge as prominent and consistent in this study. Participants described the PSD as an external regulator, one that imposed structure, demanded responsiveness, and created a sense of responsibility that mobilized them during periods of low mood, avoidance, and functional withdrawal. The mutual nature of the caregiving relationship was particularly striking: caring for the PSD appeared to reinforce self-care in handlers, creating a virtuous cycle in which attending to the dog’s needs supported adherence to basic routines such as waking, eating and exercising that are commonly disrupted by psychiatric illness. This is consistent with prior qualitative work suggesting that PSD ownership can restore a sense of identity, purpose, and self-efficacy (18), and with broader evidence from the companion animal literature that the bond between a veteran with PTSD and their adopted companion dog increased social interactions and improved emotional wellbeing (12). A study of pet ownership and mental health in older adults, identified sense of purpose and meaning, together with the imposition of routine, as among the most consistently reported mechanisms through which pet ownership benefited mental health (13). Participants in their study described how caring for a pet gave their day structure and gave them a reason to get up in the morning, language that closely mirrors the accounts of PSD handlers in the present study, who described the dog’s biological needs as an external motivational scaffold that activated self-care even when internal motivation was absent (13). Recent research has also found that having a psychiatric service dog is associated with fewer PTSD symptoms, less depression and anxiety, and better quality of life (28).These benefits appear to be due to a combination of the service dog’s specific trained tasks and aspects inherent to cohabitating with a service dog, including having a source of affection, nonjudgmental social support, and companionship (28). O’Haire et al. (2015) also noted that the social benefits of animal assisted interventions were not limited to the direct interaction between the animal and the person, but extended to improved relationships with other humans, a pattern clearly illustrated in the present data, where participants described the PSD as having rebuilt not only their capacity to engage with strangers but also their relationships with family members and friends from whom psychiatric illness had estranged them (29). This suggests that the social effects of PSD partnership should be understood not as a single mechanism but as a cascading set of processes: the dog enables social contact, which builds confidence, which enables deeper engagement, which over time rebuilds social networks.

Participants in this study also described gains in independence as a key mediator of improved social functioning. The presence of the PSD enabled handlers to access public spaces, attend social events, and complete daily activities that anxiety, avoidance, and reliance on others had previously rendered inaccessible. This increased independence was experienced not merely as practical but as psychologically meaningful, a reclamation of agency and self-determination that appears to have downstream effects on social confidence and willingness to engage. Importantly, some participants described their PSD as helping to manage the social interaction itself in public, providing a focal point for others that deflected scrutiny from the handler, and offering a learned signal that could extricate the handler from unwanted interactions. A recent mixed methods study suggested that PSDs provide benefits beyond specific trained tasks, offering ongoing support that helps handlers feel safer and more able to engage in activities of daily living and mental health treatment (30). In a survey study in the United States exploring the benefits of being teamed with a service dog, respondents agreed or strongly agreed to benefits of emotional connection, community participation, physical activity, psychological wellbeing and quality of life (31).

Participants also consistently described a range of negative experiences that created significant burden and, in some cases, directly undermined the very benefits the PSD partnership was intended to provide. Organizational leads described the need for taking a potential PSD handler’s ability to navigate access discrimination; intrusive public questioning and the involuntary disclosure of invisible disability into account before recommending partnership with a PSD. The paradoxical increase in anxiety that public attention could produce represent a dimension of PSD partnership that is largely absent from the companion animal literature, which has predominantly examined pets in private and semi-private settings rather than in the contentious public access contexts that characterise PSD use.

Several limitations of this study warrant acknowledgement. The sample was recruited via PSD training organisations, which may introduce selection bias toward handlers with more positive experiences or greater organisational support. As a qualitative study, the findings are not designed to establish causation or to be generalized statistically; rather, they illuminate the range and depth of experiences within this population. Furthermore, most participants were also receiving pharmacological and/or psychological treatment concurrently, which makes it impossible to attribute observed improvements in social functioning and loneliness exclusively to PSD partnership. Future research should seek to address these limitations through longitudinal mixed-methods designs, more linguistically and demographically diverse sampling strategies, and validated quantitative measures of loneliness and social functioning administered alongside qualitative interviews.

This study makes a meaningful contribution to the growing literature on PSDs and psychosocial well-being by providing the first qualitative investigation of the experiences of PSD handlers in South Africa across a diverse range of psychiatric diagnoses. The findings affirm the evidence that PSDs are associated with reductions in loneliness and improvements in social functioning, while importantly illuminating the mechanisms through which these benefits occur and the social costs that may accompany them. Clinically, these findings suggest that PSDs have the potential to address loneliness and social isolation as a complement to, rather than a replacement for, conventional psychiatric treatment. Mental health professionals may benefit from greater awareness of PSDs as a viable adjunctive intervention, and from understanding the range of psychosocial benefits as well as the challenges that handlers are likely to encounter.

In conclusion, for many participants in this study, the PSD partnership represented far more than a trained task-performer, they were described as a companion, a social bridge, a source of purpose, and a daily motivator that had enabled some them to re-enter social and community life after periods of withdrawal. These experiences, grounded in the specific social and clinical context of South Africa, offer a nuanced portrait of the ways in which psychiatric service dogs can address the social dimensions of psychiatric disability, and underscore the need for continued, rigorous, and internationally diverse research in this area.

## Data Availability

The raw data supporting the conclusions of this article will be made available by the authors, without undue reservation.
